# IGF-1 and ADMA Levels Are Inversely Correlated in Nondiabetic Ankylosing Spondylitis Patients Undergoing Anti-TNF-Alpha Therapy

**DOI:** 10.1155/2014/671061

**Published:** 2014-09-11

**Authors:** Fernanda Genre, Raquel López-Mejías, Javier Rueda-Gotor, José A. Miranda-Filloy, Begoña Ubilla, Aurelia Villar-Bonet, Beatriz Carnero-López, Inés Gómez-Acebo, Ricardo Blanco, Trinitario Pina, Carlos González-Juanatey, Javier Llorca, Miguel A. González-Gay

**Affiliations:** ^1^Epidemiology, Genetics and Atherosclerosis Research Group on Systemic Inflammatory Diseases, Rheumatology Division, IDIVAL, 39011 Santander, Spain; ^2^Rheumatology Division, Hospital Lucus Augusti, 27003 Lugo, Spain; ^3^Endocrinology Division, Hospital Clínico Universitario, 47005 Valladolid, Spain; ^4^Oncology Division, Hospital Del Bierzo, Ponferrada, 24411 León, Spain; ^5^Department of Epidemiology and Computational Biology, School of Medicine, University of Cantabria, IDIVAL and CIBER Epidemiología y Salud Pública (CIBERESP), 39011 Santander, Spain; ^6^Cardiology Division, Hospital Lucus Augusti, 27003 Lugo, Spain; ^7^Cardiovascular Pathophysiology and Genomics Research Unit, School of Physiology, Faculty of Health Sciences, University of the Witwatersrand, Johannesburg 2000, South Africa

## Abstract

Like rheumatoid arthritis, ankylosing spondylitis (AS) is also an inflammatory disease associated with accelerated atherosclerosis and the presence of metabolic syndrome (MeS) features. AS patients often display osteoporosis as well as new bone formation. Insulin-like growth factor 1 (IGF-1) is a protein involved in both inflammation and bone metabolism. In the present study we assessed whether disease activity, systemic inflammation, MeS features, adipokines, and biomarkers of endothelial activation were associated with IGF-1 and insulin-like growth factor binding protein-3 (IGFBP-3) levels in a series of 30 nondiabetic AS patients without CV disease undergoing TNF-*α* antagonist-infliximab therapy. All determinations were made in the fasting state, immediately before an infliximab infusion. Although no association of IGF-1 and IGFBP-3 levels with angiopoietin-2 or osteopontin was found, an inverse correlation between IGF-1 levels and asymmetric dimethylarginine (ADMA), an endogenous endothelial nitric oxide synthase inhibitor that impairs nitric oxide production and secretion promoting endothelial dysfunction, was found (*r* = −0.397; *P* = 0.04). However, no significant association was found between IGF-1 and IGFBP-3 levels and disease activity, systemic inflammation, metabolic syndrome features, or adipokines. In conclusion, in nondiabetic patients with AS undergoing periodic anti-TNF-*α* therapy, IGF-1 and ADMA are inversely correlated.

## 1. Introduction

Like rheumatoid arthritis, ankylosing spondylitis (AS) is also a chronic inflammatory rheumatic disease in which an increased incidence of cardiovascular (CV) mortality due to accelerated atherosclerosis has been reported [1]. Besides the typical manifestations of AS such as synovitis, enthesitis, uveitis, and new bone formation [2], AS patients often display a dysregulation of adipokines and metabolic syndrome (MeS) features (obesity, dyslipidemia, hypertension, and alterations in glucose metabolism, including insulin resistance (IR)) [3, 4]. Regarding therapeutic strategies for the treatment of AS, anti-TNF-*α* therapy was found to be effective to treat patients with this disease [5–7]. Anti-TNF-*α* agents lead to a suppression of inflammation and thus to a reduction of disease activity, as well as to an improvement of endothelial function in AS patients [8, 9]. This is the reason why the assessment of new potential CV risk biomarkers and the influence of anti-TNF-*α* therapy on them could shed light on the biologic mechanisms of these biologic agents associated with atherosclerosis in AS patients.

Our group has previously evaluated the involvement of metabolic syndrome (MeS) related biomarkers, adipokines, and biomarkers of endothelial cell activation and inflammation in a series of nondiabetic AS patients on periodical treatment with the anti-TNF-*α* monoclonal antibody, infliximab. Regarding MeS related biomarkers, we disclosed a link between IR and serum ghrelin concentration in our series of AS patients [10], as well as an association between retinol binding protein-4 (RBP-4) and MeS features such as IR and systolic blood pressure [11]. We also assessed the potential association between different adipokines and clinical and demographic features of AS. In this regard, we found a positive correlation between adiponectin serum levels and insulin sensitivity (IS), suggesting that low circulating adiponectin concentrations may be associated with metabolic abnormalities that promote CV disease in AS [12]. We also observed an association between adiponectin levels and the presence of involvement or synovitis and/or enthesitis in other peripheral joints [12]. Finally, we disclosed a correlation between visfatin levels and IR [13]. With regard to biomarkers of endothelial cell activation and inflammation, we observed a link between asymmetric dimethylarginine (ADMA) concentration and some features of MeS [14], an association between angiopoietin-2 (Angpt-2) serum levels and the age at the onset of symptoms of AS and disease duration [15], and also a positive correlation between serum levels of osteopontin (OPN) and Angpt-2 [16]. Furthermore, we also disclosed an independent correlation between osteoprotegerin (OPG) and ADMA [17] and an inverse correlation between TNF-related apoptosis-inducing ligand (TRAIL) and IS and resistin [18].

Insulin-like growth factor 1 (IGF-1) is produced by many tissues, mainly by the liver, and it is involved in biologic processes such as osteoblasts growth and differentiation [19]. IGF-1 is also involved in the modulation of immunity and inflammation [20]. Circulating IGF-1 levels are under the control of growth hormone (GH). In addition, IGF-1's effects are modulated by members of the insulin-like growth factor binding proteins (IGFBP) [21]. Insulin-like growth factor binding protein-3 (IGFBP-3) is a 264-amino acid peptide produced by the liver. It is the most abundant of a group of IGFBP that transport and control bioavailability and half-life of IGF, in particular IGF-1 [21].

Even if new bone formation is a typical feature of AS, these patients can also display bone loss, leading to an increased rate of vertebral compression fractures [22]. In this regard, IGF-1 has been associated with osteoporosis, being reported decreased levels of this protein in patients with AS [23, 24]. In line with this, Johansson et al. showed that IGF-1 administration promoted bone formation in osteoporotic patients [25]. Regarding inflammation, previous studies have shown that IGF-1 is inversely correlated to erythrocyte sedimentation rate (ESR), a marker of inflammation [26].

Taking all these considerations into account, in the present study we aimed to assess potential associations between disease activity, systemic inflammation, adipokines and biomarkers of endothelial activation, and MeS features with circulating IGF-1 and IGFBP-3 levels in nondiabetic AS patients undergoing infliximab therapy.

## 2. Patients and Methods

### 2.1. Patients

We assessed a series of 30 patients with AS attending hospital outpatient clinics seen over 14 months (January 2009 to March 2010), who fulfilled the modified New York diagnostic criteria for AS [27]. They were treated by the same group of rheumatologists and were recruited from the Hospital Lucus Augusti (Xeral-Calde), Lugo, Spain.

AS patients on treatment with infliximab seen during the period of recruitment with diabetes mellitus or with plasma glucose levels greater than 110 mg/dL were excluded. None of the patients included in the study had hyperthyroidism or renal insufficiency. Also, patients seen during the recruitment period who had experienced CV events, including ischemic heart disease, heart failure, cerebrovascular accidents, or peripheral arterial disease, were excluded. Patients were diagnosed as having hypertension if blood pressure was >140/90 mmHg or they were taking antihypertensive agents. Patients were considered to have dyslipidemia if they had hypercholesterolemia and/or hypertriglyceridemia (defined as diagnosis of hypercholesterolemia or hypertriglyceridemia by the patients' family physicians, or total cholesterol and/or triglyceride levels in fasting plasma were >220 mg/dL and >150 mg/dL, resp.). Obesity was defined if body mass index (BMI) (calculated as weight in kilograms divided by height in squared meters) was greater than 30.

In all cases treatment with the anti-TNF-*α* monoclonal antibody, infliximab, was started because of active disease. All patients included in the current study had begun treatment with NSAIDs immediately after the disease diagnosis. All of them were still being treated with these drugs at the time of the study. At the time of this study most patients were on treatment with naproxen: 500–1000 mg/d. Although the 2010 updated recommendations facilitate initiation of TNF-*α* blockers in AS and only ask for 2 NSAIDs with a minimum total treatment period of 4 weeks [28], for the initiation of anti-TNF-*α* therapy in these series of patients recruited between January 2009 and March 2010, they had to be treated with at least 3 NSAIDs prior to the onset of infliximab therapy.

A clinical index of disease activity (Bath ankylosing spondylitis disease activity index—BASDAI—range of 0 to 10) [29] was evaluated in all patients at the time of the study. Clinical information on hip involvement, history of synovitis in other peripheral joints and peripheral enthesitis, history of anterior uveitis, presence of syndesmophytes, and HLA-B27 status (typed by cell cytotoxicity) was assessed. Moreover, C-reactive protein (CRP) by a latex immunoturbidity method, ESR-Westergren, serum glucose, total cholesterol, high-density lipoprotein (HDL) cholesterol and low-density lipoprotein (LDL) cholesterol, and triglycerides (fasting overnight determinations) were assessed in all the patients at the time of the study.

The characteristics of the AS patients included in this study have been previously described [18]. Since at that time all patients were undergoing periodical treatment with the anti-TNF-*α* monoclonal antibody, infliximab (median duration of periodical treatment with this biologic agent: 23 months), the mean BASDAI ± standard deviation (SD) was only 2.94 ± 2.11.

The local institutional committee approved anti-TNF-*α* therapy. Also, patients gave informed consent to participate in this study. This study was not supported by a pharmaceutical drug company.

### 2.2. Study Protocol

All determinations were made in the fasting state, prior to an infliximab infusion. Blood samples were taken at 0800 hours for determination of the ESR (Westergren), CRP (latex immunoturbidimetry), lipids (enzymatic colorimetry), plasma glucose, and serum insulin (DPC, Dipesa, Los Angeles, CA, USA). As previously described, insulin resistance was estimated by the homeostasis model assessment of insulin resistance (HOMA-IR) using the formula insulin (*μ*U/mL) × glucose (mmoL/L) ÷ 22.57 [30]. A commercial ELISA kit was used to measure plasma IGF-1 and IGFBP-3 levels (R&D Systems, DG100 and DGB300; assay sensitivity = 0.026 and 0.05 ng/mL; intra- and interassay coefficients of variation were <4% and <8% for IFG-1 ELISA kit and <4% and <6.6% for IFGBP-3 ELISA kit, resp.) (Abingdon, UK) according to the manufacturer's instructions. Total plasma adiponectin and OPG levels, serum resistin, leptin, visfatin, apelin, Angpt-2, ADMA, gelsolin, ghrelin, OPN, RBP-4, and TRAIL levels were determined by ELISA as previously described [10–18, 31, 32].

### 2.3. Statistical Analyses

Variables were expressed as mean ± SD or percentages. Correlation between IGF-1 and IGFBP-3 plasma levels with selected continuous variables was performed adjusting for age at the time of the study, sex, and classic CV risk factors via estimation of the Pearson partial correlation coefficient (*r*).

The associations between characteristics and plasma IGF-1 and IGFBP-3 concentrations were assessed by Student's paired *t*-test. Differences in IGF-1 and IGFBP-3 levels between men and women and patients with hypertension or not were assessed by Mann-Whitney *U* test.

Two-sided *P* values ≤0.05 were considered to indicate statistical significance. Analyses were performed using Stata 12/SE (StataCorp, College Station, TX).

## 3. Results

### 3.1. Relationship of IGF-1 and IGFBP-3 Levels with Disease Activity and Clinical Features

No difference was disclosed between circulating IGF-1 and IGFBP-3 and disease activity parameters, such as disease duration, BASDAI, or VAS spinal pain at the time of the study (Table 1). Similarly, we did not observe any difference in IGF-1 or IGFBP-3 levels when patients were stratified according to history of anterior uveitis, presence of syndesmophytes, hip involvement or synovitis in other peripheral joints, peripheral enthesitis, and HLA-B27 status (data not shown).

### 3.2. Relationship of Demographic Features, Inflammation, Adiposity, and Adipokines with Circulating IGF-1 and IGFBP-3 Levels

We did not observe any significant association between IGF-1 and IGFBP-3 plasma levels and age at the onset of symptoms, BMI, CRP, or ESR at the time of the study and at the time of disease diagnosis (Table 1). Likewise, no association with any of the adipokines studied was observed (Table 2). Additionally, no difference in IGF-1 and IGFBP-3 concentration between men and women was observed (data not shown).

### 3.3. Relationship of IGF-1 and IGFBP-3 Levels with MeS Features Other Than Adiposity

IGF-1 and IGFBP-3 plasma levels did not show any statistical correlation with systolic or diastolic blood pressure, total cholesterol, LDL cholesterol, HDL cholesterol, triglycerides, serum glucose levels, insulin sensitivity (QUICKI), or insulin resistance (HOMA-IR) (Tables 1 and 2). Similarly, we did not find a correlation between IGF-1 and IGFBP-3 concentration and MeS-associated biomarkers such as ghrelin or RBP-4 (Table 2). Besides, when patients were stratified according to the presence or absence of arterial hypertension, no significant differences in IGF-1 and IGFBP-3 plasma levels were seen (not shown).

### 3.4. Relationship of IGF-1 and IGFBP-3 Plasma Levels with Biomarkers of Endothelial Cell Activation and Atherosclerosis

We found an inverse correlation between IGF-1 and ADMA levels (*r* = −0.397; *P* = 0.04) (Table 2). However, no association was observed between IGFBP-3 and ADMA levels. Likewise, no correlation was observed between IGF-1 and IGFBP-3 and Angpt-2 or OPN (Table 2).

## 4. Discussion

The present study discloses an inverse correlation between levels of IGF-1 and ADMA, a biomarker of endothelial cell activation, in AS patients under periodic treatment with anti-TNF-*α* therapy. Our results are in keeping with those obtained by Setola et al., who also reported that IGF-1 and ADMA levels were negatively associated after six months of growth hormone treatment [33].

Previous* in vivo* studies demonstrate that IGF-1 stimulation induces an increase in endothelial nitric oxide synthase (eNOS) activity, with the ensuing increase of nitric oxide (NO) [34]. Taking into account that NO is a key factor for the maintenance of vascular homeostasis, this could lead to an improvement of endothelial function. In contrast, ADMA (an endogenous eNOS synthase inhibitor) impairs NO production and secretion [14]. Consequently, increased levels of ADMA could be detrimental for endothelial function and have indeed been associated with subclinical markers of atherosclerosis and cardiovascular disease [35, 36]. In accordance with these data, Ji et al. recently performed a study to assess the potential relationship between vascular endothelial function in hypercholesterolemic patients and serum IGF-1 and ADMA levels. They found that IGF-1 levels positively correlated with flow-mediated arterial diastolic function (FMD), while ADMA levels showed a negative correlation with FMD [37]. These results further support the idea that IGF-1 is involved in vascular endothelial function.

In previous studies performed in our cohort of AS patients undergoing anti-TNF-*α* therapy, we found that ADMA levels were associated with features of MeS, such as hypertension [14]. Even if in the present study we did not observe any correlation between IGF-1 and MeS features, we did find an inverse correlation of IFG-1 and ADMA. Probably, as a result of long-term anti-TNF-*α* treatment (median = 23 months), ADMA levels were decreased in our AS patients, while IGF-1 levels were increased. In keeping with these observations, an inverse correlation between TNF-*α* and IGF-1 levels was disclosed in AS patients by Lange et al. [38]. In addition, Briot et al. showed an increase in IGF-1 levels in AS patients being treated with infliximab [39]. Therefore, this could be a potential mechanism by which anti-TNF-*α* therapy improves endothelial function in AS patients.

It is important to highlight that this is a well-controlled population, displaying low disease activity levels at the time of the study as a result of long-term anti-TNF-*α* therapy [8]. Therefore, this may explain the lack of association of IGF-1 and IGFBP-3 with most of the variables included in this study.

## 5. Conclusion

We can conclude that, in nondiabetic patients with AS undergoing periodic anti-TNF-*α* therapy, IGF-1 and ADMA are inversely correlated.

## Figures and Tables

**Table 1:** Partial correlation of plasma IGF-1 and IGFBP-3 with clinical characteristics and routine laboratory parameters prior to an infliximab infusion, adjusting by age at the time of the study, sex, and classic cardiovascular risk factors (dyslipidemia, smoking, obesity, and hypertension) in 30 nondiabetic patients with ankylosing spondylitis.

Variable	IGF-1		IGFBP-3	
	*r*	*P*	*r*	*P*
Clinical characteristics				
Age at the onset of symptoms	−0.274	0.17	0.111	0.58
Disease duration∗	0.353	0.07	−0.029	0.89
BMI∗	−0.032	0.87	−0.191	0.34
Systolic blood pressure∗	0.245	0.22	0.196	0.33
Diastolic blood pressure∗	−0.012	0.95	0.192	0.34
BASDAI∗	−0.044	0.83	−0.006	0.98
VAS spinal pain∗	−0.119	0.55	0.169	0.40
Routine laboratory parameters				
ESR∗ (natural-log-transformed)	−0.104	0.61	0.174	0.39
CRP∗ (natural-log-transformed)	−0.045	0.82	−0.111	0.58
ESR∗∗ (natural-log-transformed)	−0.033	0.87	0.108	0.59
CRP∗∗ (natural-log-transformed)	−0.008	0.97	−0.132	0.51
Total cholesterol∗ (natural-log-transformed)	−0.030	0.88	0.020	0.92
HDL cholesterol∗ (natural-log-transformed)	−0.102	0.61	−0.138	0.49
LDL cholesterol∗ (natural-log-transformed)	0.071	0.73	0.041	0.84
Atherogenic index∗ (total cholesterol/HDL)	0.102	0.62	0.161	0.43
Triglycerides∗ (natural-log-transformed)	−0.084	0.68	0.173	0.39
Serum glucose∗ (natural-log-transformed)	0.054	0.79	−0.225	0.26

*At the time of the study. **At the time of disease diagnosis.

BASDAI: Bath ankylosing spondylitis disease activity index; BMI: body mass index; CRP: C-reactive protein; ESR: erythrocyte sedimentation rate; HDL: high-density lipoprotein; IGF-1: insulin-like growth factor 1; IGFBP-3: insulin-like growth factor binding protein-3; LDL: low-density lipoprotein; VAS: visual analogue scale.

**Table 2:** Partial correlation of plasma IGF-1 and IGFBP-3 with metabolic parameters at baseline, prior to an infliximab infusion, adjusting by age at the time of the study, sex, and classic cardiovascular risk factors (dyslipidemia, smoking, obesity, and hypertension) in 30 nondiabetic patients with ankylosing spondylitis.

Variable	IGF-1		IGFBP-3	
	*r*	*P*	*r*	*P*
HOMA-IR at time 0∗	0.273	0.17	0.052	0.80
QUICKI at time 0∗	−0.280	0.16	−0.116	0.56
Resistin at time 0	0.341	0.12	−0.270	0.22
Adiponectin at time 0	−0.215	0.29	0.158	0.44
Leptin at time 0	0.313	0.12	−0.310	0.12
Visfatin at time 0	−0.074	0.72	0.068	0.74
Angpt-2 at time 0	0.080	0.69	0.274	0.17
Apelin at time 0	−0.005	0.98	0.237	0.23
**ADMA at time 0**	**−0.397**	**0.04**	−0.346	0.08
Ghrelin at time 0	−0.175	0.39	−0.327	0.10
Gelsolin at time 0	0.148	0.46	0.180	0.37
OPN at time 0	0.168	0.40	0.004	0.99
RBP-4 at time 0	0.339	0.08	0.053	0.79
OPG at time 0	−0.049	0.81	0.063	0.76
TRAIL at time 0	0.109	0.59	0.122	0.54
IGFBP-3 at time 0	0.163	0.42	—	—
IGF-1 at time 0	—	—	0.163	0.42

*At the time of the study.

ADMA: asymmetric dimethylarginine; Angpt-2: angiopoietin-2; HOMA-IR: homeostasis model assessment of insulin resistance; IGF-1: insulin-like growth factor 1; IGFBP-3: insulin-like growth factor binding protein-3; OPG: osteoprotegerin; OPN: osteopontin; QUICKI: quantitative insulin sensitivity check index; RBP-4: retinol binding protein-4; TRAIL: TNF-related apoptosis-inducing ligand. Significant results are highlighted in bold.
